# Synergetic Effects of Aloe Vera Extract with Trimethylglycine for Targeted Aquaporin 3 Regulation and Long-Term Skin Hydration

**DOI:** 10.3390/molecules29071540

**Published:** 2024-03-29

**Authors:** Viktor Filatov, Anna Sokolova, Natalya Savitskaya, Mariya Olkhovskaya, Andrey Varava, Egor Ilin, Elizaveta Patronova

**Affiliations:** 1 Science Center, SkyLab AG, 1066 Lausanne, Switzerlandresearch@skylaboratory.ch (N.S.); patronova@splat.ru (E.P.); 2 Department of Pharmaceutical Chemistry and Organization of Pharmaceutical Business, Faculty of Basic Medicine, Lomonosov Moscow State University, Moscow 119991, Russia; 3 Faculty of Chemistry, Lomonosov Moscow State University, Moscow 119991, Russia; 4 N. D. Zelinsky Institute of Organic Chemistry, Moscow 119991, Russia

**Keywords:** aquaporin, phytochemicals, synergism, moisturizing effect, skin, epidermis, extracts

## Abstract

Aquaporin 3 (AQP3) channels are tetrameric membrane-bound channels that facilitate the transport of water and other small solutes across cell membranes in the skin. Decreased AQP3 expression is associated with skin dryness, skin aging, psoriasis, and delayed wound healing. Thus, our study focused on a novel combination based on *Aloe barbadensis* leaf extract and trimethylglycine for targeted AQP3 regulation in skin keratinocytes and deep skin moisturization. Firstly, a dose-finding cytotoxicity assay of the selected substances was performed with a 2,5-diphenyl-2H-tetrazolium bromide (MTT) indicator on HaCaT cells. The substances’ ability to increase the amount of AQP3 in keratinocytes was evaluated in a keratinocyte cell culture by means of ELISA. Additionally, the deep skin hydration effect was confirmed in clinical research with healthy volunteers. According to the results, the maximum tolerated doses providing viability at 70% (MTD_s_) values for *Aloe barbadensis* leaf extract and trimethylglycine were 24.50% and 39.00%, respectively. Following the research and development, a complex based on *Aloe barbadensis* leaf extract and trimethylglycine in a 1:1 mass ratio exhibited a good cytotoxicity profile, with an MTD_s_ value of 37.90%. Furthermore, it was shown that the combination had a clear synergetic effect and significantly increased AQP3 by up to 380% compared to the negative control and glyceryl glucoside (*p* < 0.001). It was clinically confirmed that the developed shower gel containing *Aloe barbadensis* leaf extract and trimethylglycine safely improved skin hydration after one use and over 28 days. Thus, this novel plant-based combination has promising potential for AQP3 regulation in the skin epidermis and a role in the development of dermatological drugs for the treatment of skin xerosis and atopic-related conditions.

## 1. Introduction

In the last few years, concern about skin xerosis has increased. The growing stress and tension caused by the global COVID-19 epidemic and other local events, the consequences of wearing medical masks and other personal protective equipment, and the constant use of sanitizers are forcing people to select personal care products carefully [1,2].

The epidermis is a five-layered skin tissue. Epidermis cells are constantly dividing and moving to the surface where the corneal layer forms. The barrier function of the skin, as well as the skin microbiota composition, which maintains the pH balance, skin hydration and moisture, is provided by this layer, as epidermis integrity damage results in excessive transepidermal water loss (TEWL) and decreased skin elasticity [3].

Aquaporins (AQPs), or “water channels”, are integral membrane proteins that serve as water transfer channels. AQPs selectively provide water molecules to the epidermal cells and allow molecules to leave the cells. The discovery of AQPs by Peter Agre and his colleagues introduced the study of AQP expression regulation as a promising target for the development of new drugs and molecules [4].

The protein structure of aquaporins, like a cylinder channel, allows water molecules to pass through it. Aquaporin 3 (AQP3) is the specific protein channel in epidermal cells supplying water to cells for skin hydration, elasticity, and skin renewal. If the AQP3 amount is decreased in skin cells, the skin becomes dry, sensitive, irritated, and damaged. Moreover, this is caused by insufficient moisture or skin barrier damage, such as peeling, different types of dermatitis, excessive mechanical exposure to scrub cleansing or cleaning with a hard washcloth, and reaction to some synthetic surfactants [5,6].

Normal expression of AQP3 is necessary for normal transepidermal water flow in the epidermal cells, TEWL decrease, skin barrier protection, wound healing, and sufficient skin elasticity. Various external and internal factors influence the AQP3 amount in epidermal cells: pH [7], heavy metal content [8], bivalent metal cations [9], and cell membrane tension [10]. There is a connection between the AQP3 amount in epidermis cells and various dermatological diseases, such as atopic dermatitis [11], psoriasis [12], vitiligo [13], and chronic skin pruritus [14]. Additionally, the decrease in AQP3 is caused by an excessive immunity reaction during the development of psoriasis [15]. In the case of insufficiently moisturized skin, there is an increased expression of AQP3; however, it is short-term and not adaptive for the long term [16]. Scientists found that increased AQP3 expression in epidermal cells promotes speeding up of the wound-healing process due to water and glycerin delivery and migration of the keratinocytes [17]. Aging significantly influences the expression level of AQP3 in the skin, decreasing the skin hydration and AQP3 expression level in the skin epidermis [5,18]. Stimulation of AQP3 expression is a potential mechanism to prevent and treat the above dermatological diseases.

The application of components that regulate the genetically embedded mechanisms of hydration and the skin moisturizing effect without damaging the skin barrier function is the most promising field in cosmetology and dermatology.

*Aloe vera* (*Aloe barbadensis*) leaf extract is known for soothing skin and relieving local inflammation. These properties are the result of a wide range of biologically active substances in the leaf extract [19]. The significant anti-inflammatory activity of *Aloe barbadensis* extract is due to an enzyme called bradykinase [20]. Aloin and vitamins C and E found in *Aloe barbadensis* leaf extract can protect the skin from the harmful effects of ultraviolet radiation (UVB), pollution from the environment, and various chemical irritants [21]. Phytosterols from *Aloe barbadensis* increase collagen synthesis and skin elasticity and improve the appearance of the skin due to estrogen-like action on ERBα and β receptors of the fibroblasts of the hand skin dermis [22]. *Aloe barbadensis* also contains several compounds that inhibit the growth of fungi, viruses, and bacteria [23]. 

N-trialkylated glycine derivative, also called trimethylglycine or betaine, is a water-soluble compound derived from sugar beet roots (*Beta vulgaris*) [24]. Trimethylglycine makes the upper skin layers, particularly the epidermic corneal layer, softer and more elastic due to its water-binding properties. Osmolytes including trimethylglycine maintain intracellular conditions for optimal metabolic activity and protect macromolecular structures from osmotic stress by regulating osmotic processes [25]. Keratinocytes are often under hyperosmotic stress, especially in skin xerosis. A direct consequence of hyperosmotic stress is the outflow of water and subsequent cell contraction. To restore cell hydration, increased absorption of osmolytes is needed while the skin condition improves from being dry to being normal and well-hydrated. Trimethylglycine is an important epidermal osmolyte, along with inositol and taurine [26]. Moreover, trimethylglycine protects protein and deoxyribonucleic acid molecules (DNA) from the action of negative factors, allowing epidermal cells to function longer for skin health and appearance [27].

Novel substances and their combinations that could increase the expression of this protein are currently being sought. Thus, the aim of this research was the development of a novel combination and evaluation of its biological activity for targeted AQP3 regulation in skin keratinocytes. According to in silico computational modeling to predict the affinity of molecules for APQ3, a combination of *Aloe barbadensis* leaf extract and trimethylglycine can stimulate AQP3 expression and the redistribution of transepidermal water flow to reduce TEWL in all layers of the epidermis. The detailed results of studies of a novel combination based on *Aloe barbadensis* leaf extract and trimethylglycine confirm the hypothesis about the increased expression of AQP3 and the optimal amount of water in the epidermis cells achieved by this targeted activity.

## 2. Results and Discussion

Any shift from the normal level of skin hydration causes not only feelings of overall discomfort but also a disruption of the deep processes that maintain skin viscoelasticity, flexibility, and skin renewal that can be crucial in the pathogenesis of dermatological disorders such as atopic dermatitis, eczema, and psoriasis [28,29]. This research reflects a full evaluation of skin hydration in silico, in vitro, and in vivo.

### 2.1. Molecular Docking of the Phytochemicals with AQP3 In Silico

**Figure 1:**
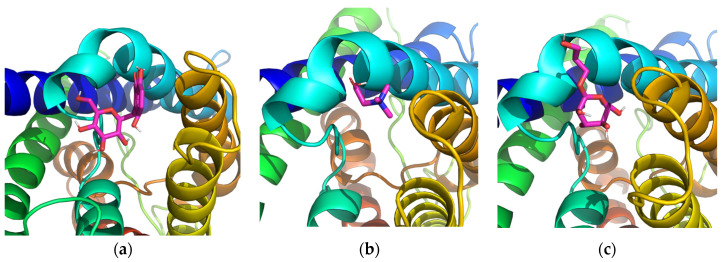
Docking visualizations of AQP3 with (**a**) aloin from *Aloe barbadensis* leaf extract; (**b**) trimethylglycine; (**c**) glyceryl glucoside.

Trimethylglycine (Figure 1b) and aloin from *Aloe barbadensis* leaf extract (Figure 1a) were selected as possible candidates for influence on AQP3 using AutoDock computational modeling (version 4.2). These natural substances have an estimated affinity score ranging from −6.2 to −7.7 kcal/mol, indicating a strong binding with the AQP3 active site. With trimethylglycine acting as a natural osmolyte, it may be possible to enhance the affinity of plant biomolecules and stabilize the structure of AQP3. As a positive control, glyceryl glucoside (Figure 1c) exhibited a mild affinity with a docking score of −4.0 kcal/mol.

### 2.2. Prediction of Pharmacological Activity of the Phytochemicals for Skin Health

Phyto4Health modeling was used to predict the pharmacological activities of aloin from the *Aloe barbadensis* leaf extract and trimethylglycine. Prediction of activity spectra for biologically active substances (PASS) was performed to estimate the possible effects during the in silico analysis. It was observed that these molecules have anti-psoriatic, antioxidant, anti-inflammatory, and immunosuppressant properties useful for the treatment of skin atopic diseases [17]. The main biological activities useful for the treatment of skin atopic diseases and skin hydration level are presented in Table 1. The probability “to be active” (Pa) is a crucial criterion to evaluate the chance that selected biologically active compounds have a pharmacological activity according to the chemical structure and PASS training set.

### 2.3. In Vitro Assessment of Maximum Tolerated Dose for the Phytochemicals

In the first stage, the cytotoxic activity of the components of the novel combination based on *Aloe barbadensis* leaf extract and trimethylglycine was estimated based on their concentration in the composition.

In this study, the 2,5-diphenyl-2H-tetrazolium bromide (MTT) assay was performed to assess the influence of the bioactive agents on skin keratinocyte cell growth and viability. In a preliminary test, the components of the composition, individually and in combination, were found to be well tolerated by skin keratinocytes. The allowed maximum tolerated doses providing viability at 70% (MTDs) of HaCaT cells were estimated to be 24.5%, 39.0%, and 37.9% when treated with *Aloe barbadensis* leaf extract, trimethylglycine, and their combination, respectively.

Based on the initial cytotoxicity assessment, HaCaT cells treated with *Aloe barbadensis* leaf extract and trimethylglycine within the required concentration range of 0.03–10.00 wt.% did not show evidence of significant cell toxicity. This concentration range is allowed for leave-on and rinse-off skin and hair care formulations. The viability of skin keratinocytes exposed to bioactive agents in this concentration range remained above the threshold value of 70%, indicating a gentle influence on the skin keratinocytes (Table 2).

With respect to the combination of *Aloe barbadensis* leaf extract and trimethylglycine in a 1:1 mass ratio, the increased viability of the skin keratinocytes was found in the entire concentration range of 0.03–10.0 wt.% compared to *Aloe barbadensis* leaf extract alone. *Aloe barbadensis* leaf extract synergized with trimethylglycine in increasing keratinocyte viability, indicating a more favorable effect of the combination on the metabolic activity of skin cells. Both bioactive substances and their combination were well tolerated by keratinocytes. Consistent with previous studies, the organic osmolyte betaine provided cell protection by acting as a “chemical chaperone”, thus providing a gentler overall effect of the composition [30,31].

For a more detailed elucidation of the AQP3 stimulation effect, the tolerated concentrations of the components with the highest survival rate were selected according to the results of the in vitro cytotoxicity assay (Table 2).

### 2.4. Investigation of AQP3 Stimulation Effect In Vitro

**Figure 2:**
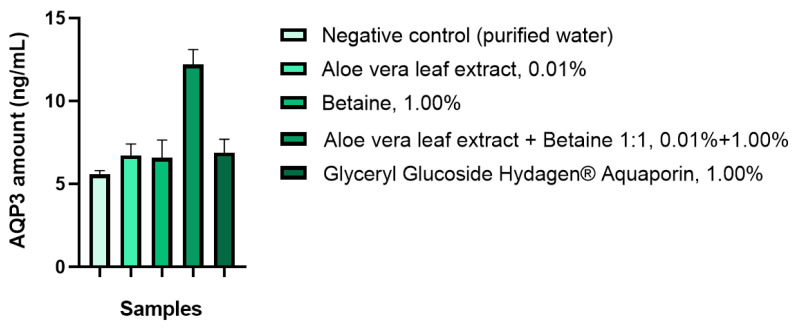
Effects of *Aloe barbadensis* leaf extract and trimethylglycine on the AQP3 amount (ng/mL) in skin epidermal cells.

Human keratinocytes were used as an in vitro model to evaluate the effect of the novel combination on the AQP3 amount in skin epidermal cells. It was revealed that the addition of the novel combination based on *Aloe barbadensis* leaf extract and trimethylglycine in a mass ratio of 1:1 equivalent to raw active ingredient amounts of 0.01 and 1.00 wt.%, respectively, to the culture medium leads to an increase in the amount of AQP3 in the skin epidermis (Figure 2).

The novel combination of *Aloe barbadensis* leaf extract and trimethylglycine in concentrations of 0.01 and 1.00 wt.%, respectively, increased the amount of AQP3 up to 12.21 ± 0.91 ng/mL, as compared to 5.58 ± 0.24 ng/mL for the negative control (Table 3). This AQP3 stimulation effect was significantly greater, by 1.77 fold, than the effect of the commercially named Hydagen^®^ Aquaporin (BASF SE, Pulnoy, France), known for its ability to influence the amount of AQP3 in skin epidermal cells.

**Figure 3:**
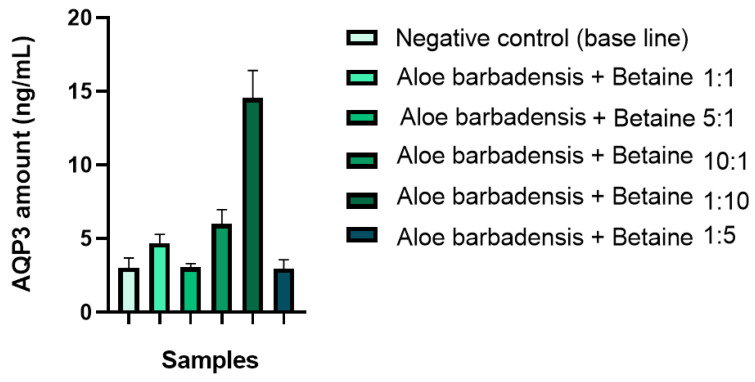
Effects of *Aloe barbadensis* leaf extract and trimethylglycine in various mass ratios and concentrations on AQP3 amount (ng/mL) in skin epidermal cells.

In addition, the combinations of *Aloe barbadensis* leaf extract and trimethylglycine in various mass ratios and concentrations were studied to select the best ratio of components for the highest influence on AQP3. When the mass concentration of trimethylglycine was fixed and the mass content of *Aloe barbadensis* leaf extract was multiplied to obtain mass ratios of 5:1 and 10:1, the highest AQP3 stimulatory effect was observed for the combination of *Aloe barbadensis* leaf extract and trimethylglycine with a mass ratio of 10:1 (Figure 3). Nevertheless, by varying the amount of trimethylglycine in the combinations, the highest increase in AQP3 level was observed for the combination of *Aloe barbadensis* leaf extract and trimethylglycine in a mass ratio of 1:10 (Figure 3). Surprisingly, the combinations of *Aloe barbadensis* leaf extract and trimethylglycine in a mass ratio of 5:1 or 1:5 did not induce changes in the AQP3 amount in skin epidermal cells in comparison with the negative control. Possibly, this unexpected result is explained by the specific interaction between the biologically active ingredients of *Aloe barbadensis* leaf extract and trimethylglycine and the formation of intramolecular complexes influencing the gene expression of AQP3 in epidermal cells [32,33]. This finding was confirmed in two independent experiments in triplicate (n = 3) according to interlaboratory precision. Thus, combinations of *Aloe barbadensis* leaf extract and trimethylglycine in specific ratios of 10:1 (equivalent to the concentrations of 0.10 wt.% + 1.00 wt.%, respectively) and 1:10 (equivalent to the concentrations of 1.00 wt.% + 0.10 wt.%, respectively) were found to synergistically increase the amount of AQP3 by +98.35% and +380.20% compared to the negative control (*p* < 0.01). This effect was higher than that of the scientifically researched combination of glycerin and glyceryl glucoside. The observed stimulatory effect of the novel plant-based combination on the AQP3 amount indicates its potential ability to improve the water flow and further enhance skin hydration.

*Aloe barbadensis* leaf extract has been used in skin care for centuries due to its moisturizing activity [31,34,35,36], while trimethylglycine provides the water-conducting properties of the skin cells, stabilizes the native structure of aquaporins in cell membranes, and makes the corneum stratum of the epidermis more elastic [25,26,27]. According to obtained experimental data, *Aloe barbadensis* leaf extract in combination with trimethylglycine increased the amount of the most abundant type of aquaporins in the skin epidermis—AQP3. The amount of AQP3 in the basal and suprabasal layers directly determines the degree of water penetration into various skin layers, the normal differentiation and peeling of keratinocytes, and the maintenance of the skin barrier layer and skin elasticity [5]. This effect may be provided by the changed gene expression of AQP3 or unidentified epigenetic mechanisms in epidermal cells [32,33]. This combination allows AQP3 to impact all layers of the epidermis, providing deep transepidermal water flow. Thus, *Aloe barbadensis* leaf extract and trimethylglycine affect skin hydration through the natural skin cell moisturizing mechanism, the discovery of which earned Peter Agre the Nobel Prize in chemistry in 2003.

### 2.5. Clinical Research of the Formulations Containing Aloe Barbadensis Leaf Extract and Trimethylglycine on Skin Hydration

The skin moisturizing activity of the combination that was observed through an increase in the AQP3 amount in vitro was confirmed in vivo. Clinical research on the skin care formulas containing the novel combination of *Aloe barbadensis* leaf extract and trimethylglycine in a specific ratio was performed to evaluate the effects on skin barrier function, TEWL, epidermal hydration, and pH balance on the skin epidermis. Healthy volunteers used the shower gel and the two-in-one shower gel in their daily routine.

**Figure 4:**
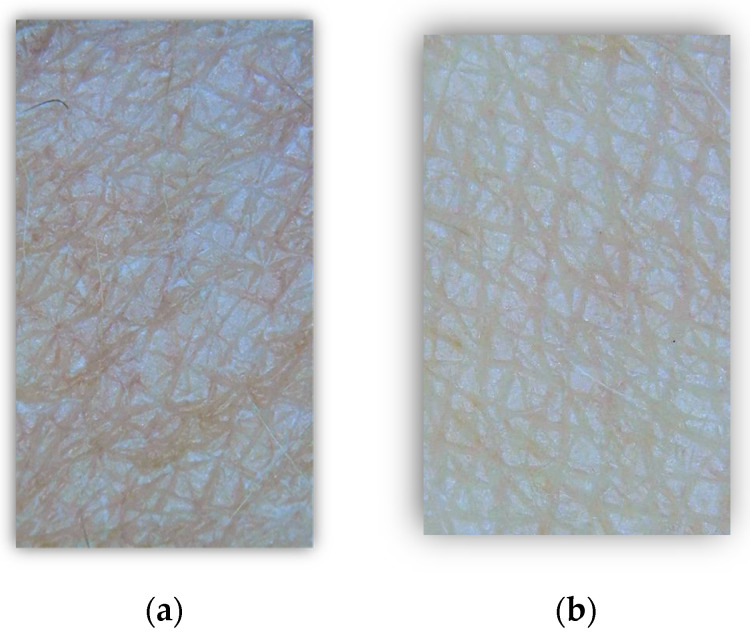
Visual instant skin hydration (**a**) before use of the shower gel with the novel combination and (**b**) 5 min after use of the shower gel with the novel combination.

The dermatological evaluation revealed an immediate increase in skin hydration 5 min after use. A strong moisturizing effect of the formulations was observed among 93% of the healthy volunteers. Epidermal hydration increased by 236% at 5 min after a single application of the shower gel, as compared to the baseline (Table 4). The formulation without the novel combination also provided skin hydration 5 min after a single application, but this observation was less statistically significant (*p* < 0.017) and was provided by the mild combination of the plant-based surfactants in the shower gel composition. The novel combination of *Aloe barbadensis* leaf extract and trimethylglycine in a specific ratio provided the moisturizing effect due to stimulation of the AQP3 in skin epidermal cells, improving the water capacity of keratinocytes and decreasing the TEWL, according to the in vitro research. The visual improvement of the skin surface was diagnosed using the ASW 300 Aramo Smart Wizard under dermatologist control (Figure 4), confirming the results in Table 4.

Skin hydration was increased after long-term application of the shower gel containing the novel combination of *Aloe barbadensis* leaf extract and trimethylglycine in a specific mass ratio. The highest skin hydration up to 25.4% was observed after using the shower gel with *Aloe barbadensis* leaf extract and trimethylglycine in a 1:10 mass ratio (Table 5), which corresponded to the results of in vitro research of the AQP3 amount. Other formulas with the active substances in a mass ratio of 1:5 to 1:6 had a mild moisturizing effect, but this beneficial effect helped to maintain the decrease in TEWL after the long-term application of body care formulas. According to the results of clinical research, more than 87% of the healthy volunteers did not observe skin tightness or dryness after long-term use of the shower gels containing the combination of *Aloe barbadensis* leaf extract and trimethylglycine.

Even small changes in skin pH play an essential role in skin system functioning. Both skin pH-dependent enzymes and microbiota are very sensitive to any changes in pH [37]. Moreover, AQP3 activity in skin cells is pH-dependent. The optimal pH value for maintaining the basic activity of AQP3 in the basal and suprabasal epidermal layers to ensure a significant amount of water and transepidermal water flow is estimated to be 5.0–7.0 [38]. Meanwhile, more acidic pH values within the physiologically optimal range promote keratinocyte differentiation and epidermis renewal, are more beneficial for maintaining normal skin microflora and the hydrolipid mantle, and effectively prevent atopic disorders in highly sensitive skin [39].

The shower gels containing the moisturizing combination of *Aloe barbadensis* leaf extract and trimethylglycine were formulated to properly regulate the pH balance of the skin epidermis. According to the results of the skin pH evaluation, the long-term use of the formulations maintained the optimal pH level of the skin from 5.06 to 5.41 with a slight shift to a more acidic area compared to the baseline level and negative control (Table 6). The observed small changes in pH contributed to more efficient water retention along with improved skin barrier function and decreased TEWL. The specific mass ratio of the *Aloe barbadensis* leaf extract and trimethylglycine in the composition of the shower gels influenced the pH index, obviously providing a beneficial effect on the skin microbiome and skin barrier function. Moreover, with an increase in the concentration of trimethylglycine, more favorable acidic pH values of the skin were observed (Table 6).

**Figure 5:**
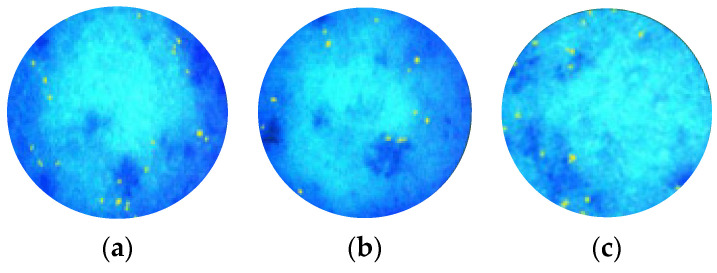
Skin sebaceous gland activity (**a**) before use of the shower gel with the *Aloe barbadensis* leaf extract and trimethylglycine combination in a mass ratio of 1:10; (**b**) 5 min after use of the shower gel; (**c**) within 28 days after use of the shower gel. The blue zones of skin mean the normal skin surface. The yellow zones reflect the active sebaceous glands in UV detection using Aramo Smart Wizard device with a lens X30-UV.

Moreover, measurements of the sebum levels and sebaceous gland activity using an imaging system revealed no influence from single or long-term use of the cleaning formulations on sebum secretion or the appearance of pores. The two-in-one shower gel with the novel combination and cleansing charcoal particles, regardless of the duration of use, did not suppress the production of sebum, allowing the body skin to maintain its skin barrier function (Table 7). The high deviation revealed the individual differences in the sebum level and genetically determined sebocyte activity, but a statistical difference was not observed after analysis. The processed images of the most active sebaceous glands with a yellow color in UV detection are presented in Figure 5. In the 28-day observation, a slight decrease in sebum production was fixed in 67% of the healthy subjects. This seboregulation helps to reduce TEWL, protects hair from mechanical damage, and maintains the biological diversity of scalp microflora to prevent dandruff, seborrheic dermatitis, and psoriasis [37].

### 2.6. Safety Assessment of the Formulations

A safety assessment of the shower gel formulations containing the novel combination of *Aloe barbadensis* leaf extract and trimethylglycine was performed before the clinical research on the skin hydration and pH level to avoid possible adverse events after widespread use of the products among consumers. The irritation and sensitization potential was evaluated in all human volunteers participating in the clinical research under dermatologist control. The safety assessment confirmed the absence of allergic reactions or irritations after a single use, 2 days, and long-term use within 28 days under dermatologist control and communication with clinical center. All tested formulations were well tolerated by participants. However, one volunteer was excluded by the dermatologist due to increased skin pH level and slight erythema before the start of the clinical research. None of the remaining participants made safety-related comments during the clinical research. None of the remaining subjects with sensitive skin had erythema, increased sensitivity, or allergic reactions. According to experimental data, the formulations containing the novel combination of *Aloe barbadensis* leaf extract and trimethylglycine in the specific ratios had good skin compatibility and can be used by people with sensitive and dry skin. Subjects’ self-assessments indicated significant satisfaction with the formulation characteristics and skin appearance.

## 3. Materials and Methods

### 3.1. Chemicals and Substances

All analytical grade chemicals used in the experiments were purchased from Sigma-Aldrich (Sigma Chemical Co., Ltd., St. Louis, MO, USA). *Aloe barbadensis* leaf extract (CAS 85507-69-3 or 94349-62-9), with the addition of water and glycerin, and trimethylglycine (CAS 107-43-7) were obtained from Bell Flavors & Fragrances GmbH (Leipzig, Germany) and IFF Health & Biosciences (Naantali, Finland), respectively.

### 3.2. Ligand and Target Preparation for Molecular Docking

**Figure 6:**
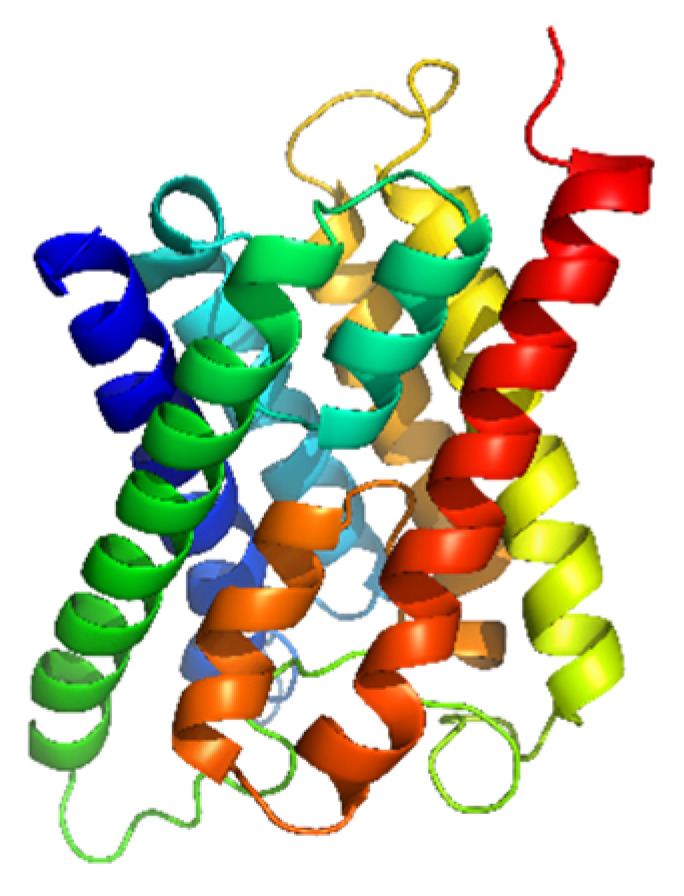
Three-dimensional structure of AQP3 from the Protein Data Bank.

The AQP3 protein (PDB ID: 3LLQ) (Figure 6) from the Protein Data Bank was used to fit the three-dimensional structure of AQP3. The protein was obtained in the special .pdb format for further docking [40]. Using AutoDock version 4.2, the protein model was prepared by eliminating water molecules, cutting out superfluous chains, and adding polar hydrogen and charges. After processing, the protein structure was saved in the .pdb and .pdbqt formats for additional in silico study.

### 3.3. Molecular Docking of Phytochemicals with AQP3

A personal computer (PC) with an Intel Core i7-12700U CPU running at 2.3 GHz and with 16 GB of RAM was used for this purpose. Windows 11, 64-bit OS, was the operating system. Firstly, the native protein ligand was used in the molecular docking process to confirm that the procedure was consistent, and the root-mean-square deviation (RMSD) was less than 2 Å. The coordinates of the grid were (X, Y, Z) 28.73, 58.834, and 63.068, and the grid box was 40 × 40 × 40. The ligand was flexible, and the macromolecule remained rigid during the docking process. AQP3 (PDB ID: 3LLQ) was docked with 2 molecules and explored using AutoDock version 4.2. The molecular docking was carried out by modifying the parameter of the genetic algorithm (GA), using ten runs of the criterion of Lamarckian GA.

### 3.4. Pharmacological Activity In Silico

The pharmacological activity was predicted using Phyto4Health (https://www.way2drug.com/p4h, accessed on 15 October 2023) [41]. This database contains information about more than 9000 phytoconstituents from different medical plants and herbs. All phytocompound structures are presented in international chemical identifier (InChI) and canonical simplified molecular-input line-entry system (SMILES) formats. This in silico program can predict the affinity of ligands to the target and pharmacological activity with approximate PASS effects and compare the physicochemical properties of molecules, such as hydrogen bond donors (HBDs), number of hydrogen bond acceptors (HBAs), number of rotatable bonds (RTBs), polar surface area (PSA), and octanol-water partition coefficient (AlogP). The Pa values are a criterion to predict the pharmacological activity for the biologically active compounds using the PASS training set.

### 3.5. Cell Culture

The human keratinocyte cell line HaCaT was purchased from MatTek (MatTek Europe, Bratislava, Slovakia). The cells were cultured in Dulbecco’s modified eagle medium (DMEM) supplemented with 5 wt.% of fetal bovine serum (FBS) in 5% CO_2_ in a humidified atmosphere at 37 °C.

### 3.6. Determination of Cytotoxic Activity

The cytotoxicity of the components on the growth and proliferation of HaCaT cells was examined by means of MTT assay by measuring the cell viability [38]. Epidermal cells were seeded in 96-well plates (96-Well EDGE Cell Culture Plates, Nest Scientific Biotechnology, Wuxi, China) at a concentration of 1 × 10^4^ cells per well and incubated for 24 h. The phytochemicals, individually or in combination, were added to the culture medium in the concentration range of 0.03–100 wt.% and incubated with the cells for 24 h. For the negative control, purified water without any components of the composition was used. After incubation with different sample dosages, cells were washed with phosphate-buffered saline (PBS) and incubated with MTT dye solution (Sigma Chemical Co., Ltd., St. Louis, MO, USA). Then, isopropanol was added to dissolve the formazan, and the absorbance was measured at 570 nm using a microplate reader (Agilent Biotek 95 spectrophotometer, Santa Clara, CA, USA). The viability of the treated cells was expressed as a percentage relative to the control. Three independent experiments were performed.

### 3.7. In Vitro Quantitative Detection of AQP3

To determine the amount of AQP3, a commercially available test using a sandwich-type enzyme-linked immunoassay was used (MatTek Europe, Bratislava, Slovakia). Cells of the HaCaT line were seeded in 96-well plates (96-Well EDGE Cell Culture Plates, Nest Scientific Biotechnology, Wuxi, China) at a concentration of 1 × 10^5^ cells per well. On the next day, the cell media were removed and replaced with fresh DMEM media with 50 µL of 5 wt.% FBS to maintain cell growth. Then, 50 µL samples of the combination were added, and cultivation was performed for 24 h. In all experiments, 0.20 wt.% sodium chloride was added to the cell medium to regulate the osmotic balance and create the physiological conditions for skin cell cultivation. The cells were cultured in 5 wt.% CO_2_ in a humidified atmosphere at 37 °C. After incubation, the cell supernatant was collected, and the amount of AQP3 in the skin keratinocytes was determined by means of ELISA. The standards and samples were added to the corresponding microplate wells with specific biotin-conjugated AQP3 antibodies. Horseradish peroxidase-conjugated avidin was then added to each microplate well and incubated. After the addition of the 3,3′,5,5′-tetramethylbenzidine (TMB) substrate solution to each microplate well, the enzyme-substrate reaction was stopped by adding a sulfuric acid solution, and the color change was measured spectrophotometrically at a wavelength of 450 ± 10 nm. The concentration of AQP3 in the sample was determined by comparing the sample with the standard curve. A physiological solution was used as the negative control. Glyceryl glucoside and glycerin (Hydagen^®^ Aquaporin BASF, BASF SE, France) were used as a positive control, known for their capacity to impact the number of aquaporins in skin cells (under the research sponsored by BASF Personal Care and Nutrition GmbH, Monheim am Rhein, Germany).

### 3.8. Body Care Formulations for the Clinical Research

Formulations containing a novel moisturizing combination of *Aloe barbadensis* leaf extract and trimethylglycine were used, as shown in Table 8.

### 3.9. Clinical Research on the Formulations Based on the Novel Composition of Aloe Barbadensis Leaf Extract and Trimethylglycine

A prospective, open-label, non-randomized, controlled clinical study was performed to evaluate the efficacy of the formulations with a novel combination based on *Aloe barbadensis* leaf extract and trimethylglycine under real conditions. In all, 60 subjects aged 18 to 62 years, with the average age being 36 ± 11.1 years, were enrolled. To estimate the efficiency, the control and three subject groups used the shower gel and the 2-in-1 shower gel twice daily (in the morning and in the evening) for 28 days. The subjects corresponded to the inclusion criteria and did not have any exclusion criteria. The study was performed in accordance with the Declaration of Helsinki after subjects signed the corresponding written consent and consulted with dermatologists.

Before the clinical research, the sensitizing and irritation potential of the formulations was assessed through a single topical application on the flexor surface of the right forearm after 2, 24, 48, and 72 h of a patch test under the control of dermatologists. In the case of a positive result, the subject used the hair and body wash under the individual hygienic routine. The average period of the study for a subject was 28 days, with 2 visits to the research center and telephone calls with a dermatologist. At the end of this stage, the subjects visited the research center to evaluate the efficacy of the shower gel formulations and answered the questionnaire about the product properties (texture, smell, foaming, cleansing, skin hydration, side effects).

**Figure 7:**
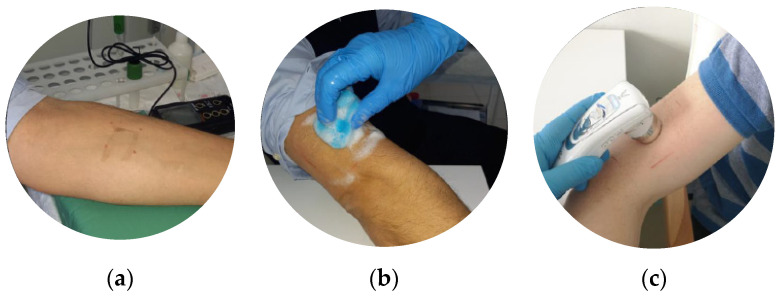
Clinical research of skin parameters using the ASW 300 device: (**a**) assessment of the irritation and sensitization potential of the formulations on the flexor surface of the right forearm; (**b**) application of the formulations during the first visit to the dermatologist on the external surface of the right shoulder; (**c**) evaluation of pH level, skin hydration, and seboregulation at different times of application on the external surface of the right shoulder.

After temperature acclimatization in the testing environment for 20–30 min at +22 ± 1 °C and air humidity of 45 ± 15%, the skin hydration effect was assessed. The moisturizing effect of the formulations containing the combination of *Aloe barbadensis* leaf extract and trimethylglycine was quantitatively evaluated using measurements made with the ASW 300 device (Aramo Smart Wizard, Medicinal Device Registration Certificate No. P3H2018/6812 dated 10 January 2020; Republic of Korea). The skin’s hydration level and its pH were estimated on the external surface of the right shoulder. The clinical procedures are presented in Figure 7.

The moisture content in all epidermal layers on the external surface of the right shoulder was determined quantitatively (%) under the conditions of electric current passage. The moisture meter was placed on the skin until the signal sounded. The measurement was carried out using a handpiece with an integrated moisture sensor. The results were analyzed in Aram Viewer software v1.1.1. (Aram Huvis Co., Ltd., Seoul, Republic of Korea).

To measure the pH of the body’s skin and scalp, a portable acid meter (HI83141 pH/мB/C-meter with HI1413B combined pH-electrode, Hanna Instruments, Vöhringen, Germany) was applied to the external surface of the right shoulder.

Measurements of skin oil and skin sebaceous gland activity were carried out using the integrated sensor of an ASW 300 device with the special lens X30-UV with UV detection. Pictures of the skin surface of right shoulder areas were analyzed in Aram Viewer software (Aram Huvis Co., Ltd., Republic of Korea). The areas of the skin with greater brightness were highlighted in yellow.

### 3.10. Safety and Irritation Assessment of the Formulations

A drop of every formulation containing the moisturizing combination of *Aloe barbadensis* leaf extract and trimethylglycine was applied to the flexor surface of the right forearm of each subject participating in the study. After 24 h, a secondary application was made to detect the sensitizing effect on a “clean” area of the skin at a distance of at least 2 cm, in the absence of subjective symptoms or objective signs of irritation. The skin reaction responses were evaluated after 2, 24, 48, and 72 h. A control skin test with distilled water was performed. To identify the sensitizing effect of the formulations and a possible delayed-type allergic reaction, a “provocative test” was carried out, starting on the 4th and extending to the 12th day. Skin reactions were assessed by dermatologists.

### 3.11. Questionnaire-Based Assessment

A diagnostic questionnaire was administered to assess consumer preferences for the formulations in their daily routine. In repeated individual interviews, subjects were asked questions about the consumer properties of the formulations in the following format: choose an answer from the proposed options, evaluate their experience on a scale from 0 to 10, or give a free-form answer. Additionally, volunteers were allowed the opportunity to leave comments about appearance, texture, smell, foaming and cleansing properties, skin hydration, and feelings of dryness and tightness.

### 3.12. Statistical Analysis

All experimental data from the in vitro study are presented as the mean values ± standard deviations (SDs) of at least three independent experiments. Differences described by *p* ≤ 0.05 were considered statistically significant after the one-way analysis of variance (ANOVA) processed using the Statistica software package (StatSoft, USA, Ver. 8.0). Otherwise, the values of different samples and controls were analyzed using non-parametric methods (Wilcoxon signed-rank test). The ELISA data normality was controlled with the Shapiro–Wilk test.

When analyzing the consumer assessment, the group mean value (M), median value (Me), and SD were calculated for all quantitative data for the control and test groups. Variables expressed as the proportion of volunteers were assessed descriptively as percentages. The data on the parameters are presented based on the nature of the variable, either continuous or categorical. Data from categorical variables were analyzed as a number (n) and proportion (percentage) of the total number of subjects having the value of the analyzed parameter. The results obtained were processed using the Statistica software package (StatSoft, Ver. 8.0). The Wilcoxon rank-sum test was used.

## 4. Conclusions

A novel combination based on *Aloe barbadensis* leaf extract and trimethylglycine was developed and investigated by means of in silico, in vitro, and clinical research. In silico modeling of the interaction of the biologically active compounds with AQP3 predicted the possible moisturizing activity and beneficial effects for skin, such as skin hydration, anti-inflammatory, and anti-eczematic atopic effects. This combination was well tolerated by keratinocytes in vitro because of the increase in cell viability in the presence of the combination. According to the in vitro experimental data, the combination of *Aloe barbadensis* leaf extract and trimethylglycine in a specific mass ratio significantly increased the amount of AQP3 in skin epidermal cells, which are proven to be involved in skin hydration, maintenance of the skin barrier, skin elasticity, normal keratinocyte differentiation, and pH balance. The AQP3 stimulation activity of the novel plant-based combination was higher than that of the single substances and commercially available glyceryl glucoside, and it was confirmed in clinical research with use by healthy volunteers. The skin formulations with this combination revealed a statistically significant improvement in instant and long-lasting skin hydration, the normalization of sebaceous gland activity, and support of the pH balance on the skin surface without negative effects. Therefore, this combination is promising for further research and the development of cosmetics and pharmaceutical products for the prophylaxis and treatment of skin xerosis, dermatitis, atopic dermatitis, psoriasis, eczema, and other dryness-related conditions.

## 5. Patents

The results of this research are part of the patent applications “Plant-mineral composition based on *Aloe barbadensis* extract and trimethylglycine for pH-dependent regulation of type 3 aquaporins and transepidermal water flow in deep epidermal layers” related to EP22217391.6.

## Figures and Tables

**Table 1:** Biological activities of aloin and trimethylglycine using the Phyto4Health database.

Compound	PASS Activities	Pa Value
Aloin from *Aloe barbadensis* leaf extract	Antioxidant	0.676
	Anti-inflammatory	0.674
	Immunosuppressant	0.524
Trimethylglycine	Anti-eczematic atopic	0.806
	Anti-psoriatic	0.570
	Antioxidant	0.259

**Table 2:** In vitro cytotoxicity assay.

Sample	Cell Viability Following the Addition of Components, wt.%				
	0.03	0.10	0.25	1.00	10.00
Negative control (purified water)	100 ± 0.00	100 ± 0.00	100 ± 0.00	100 ± 0.00	100 ± 0.00
*Aloe barbadensis* leaf extract	83.2 ± 2.25	82.7 ± 3.04	78.5 ± 6.15	80.0 ± 0.66	72.0 ± 4.03
Trimethylglycine	98.8 ± 3.47	99.9 ± 1.47	95.4 ± 2.94	93.5 ± 1.66	90.3 ± 6.82
*Aloe barbadensis* leaf extract + Trimethylglycine in a 1:1 ratio	95.4 ± 5.81	99.3 ± 4.15	99.1 ± 6.50	99.3 ± 16.80	86.9 ± 7.81

All data are presented as mean values ± standard deviation (n = 3).

**Table 3:** The effects of compounds on AQP3 expression in skin epidermal cells.

Sample	AQP3, ng/mL	AQP3 Expression Change Compared with the Negative Control, %
Negative control (purified water)	5.58 ± 0.24	-
*Aloe barbadensis* leaf extract, 0.01 wt.%	6.73 ± 0.69 *	+20.61% *
Trimethylglycine, 1.00 wt.%	6.58 ± 1.08	+17.92%
*Aloe barbadensis* leaf extract + Trimethylglycine, 0.01 wt.% + 1.00 wt.% respectively	12.21 ± 0.91 **	+118.82% **
Glycerin and Glyceryl Glucoside (Hydagen^®^ Aquaporin), 1.00 wt.%	6.89 ± 0.82 *	+23.48% *

All experiments were performed in quadruplicate. Significance levels according to statistical analysis, calculation of the mean values ± standard deviation, and comparison for different samples by non-parametric methods (Wilcoxon signed-rank test). * *p* < 0.05; ** *p* < 0.01.

**Table 4:** Skin hydration after a single application of the formulas with and without the novel combination.

Area	Formulation	Novel Combination	Skin Moisture Index by Measuring Coefficient of Keratinocyte Conductivity, %		
			Baseline	5 min after Single Application	Relative Change of Skin Moisture Index Compared with the Baseline, %
Body skin	Shower gel	Negative control (without the novel combination)	9.67 ± 4.98	17.80 ± 11.87 *	+84.0% *
		1.0 wt.% Trimethylglycine + 0.1 wt.% *Aloe barbadensis* leaf extract		32.53 ± 14.88 **	+236.0% **

Significance levels: * *p* < 0.05; ** *p* < 0.01 after statistical analysis using Statistica StatSoft Ver. 8.0.

**Table 5:** Skin hydration after long-term use of the formulas with and without the novel combination.

Area	Formulation	Novel Combination	Skin Moisture Index by Measuring Coefficient of Keratinocyte Conductivity, %		
			Baseline	28 Days of Regular Use	Relative Change of Skin Moisture Index Compared with the Baseline, %
Body skin	Shower gel	Negative control (without the novel combination)	23.5 ± 2.3	23.7 ± 2.3	+0.9%
		0.5 wt.% Trimethylglycine + 0.1 wt.% *Aloe barbadensis* leaf extract	21.3 ± 2.1	22.8 ± 2.2	+7.1%
		0.6 wt.% Trimethylglycine + 0.1 wt.% *Aloe barbadensis* leaf extract	20.1 ± 2.2	21.2 ± 2.3	+5.5%
		1.0 wt.% Trimethylglycine + 0.1 wt.% *Aloe barbadensis* leaf extract	25.2 ± 2.5	31.6 ± 3.1 *	+25.4% *

Significance level: * *p* < 0.05 after statistical analysis using Statistica StatSoft Ver 8.0.

**Table 6:** Evaluation of the pH level of the skin for different times of application.

Area	Formulation	Novel Combination	pH Index		
			Baseline	28 Days of Regular Use	Relative Change of pH Index Compared with the Baseline, %
Body skin	Shower gel	Negative control (without the novel combination)	5.68 ± 0.92	5.68 ± 0.78	0%
		0.5 wt.% Trimethylglycine + 0.1 wt.% *Aloe barbadensis* leaf extract		5.41 ± 0.59	−4.7%
		1.0 wt.% Trimethylglycine + 0.1 wt.% *Aloe barbadensis* leaf extract		5.40 ± 0.62	−4.9%
		2.0 wt.% Trimethylglycine + 0.1 wt.% *Aloe barbadensis* leaf extract		5.06 ± 0.40 *	−9.2% *

Significance level: * *p* < 0.05 after statistical analysis using Statistica StatSoft Ver 8.0.

**Table 7:** Evaluation of the sebocyte activity of the skin for different application times.

Area	Formulation	Novel Combination	Sebocyte Activity Index		
			Baseline	in 5 min after Use	28 Days of Regular Use
Body skin	2-in-1 shower gel	0.5 wt.% Trimethylglycine + 0.1 wt.% *Aloe barbadensis* leaf extract	27.33 ± 18.57	24.40 ± 22.97	25.86 ± 17.73

Significance level: *p* < 0.05 after statistical analysis using Statistica StatSoft Ver 8.0 (USA).

**Table 8:** The formulations tested in the clinical research.

Main Compounds in Formulations	Concentrations in the Formulations, wt.%		
	2-in-1 Shower Gel for Body and Hair	Shower Gel for Body	Shower Gel for Sensitive Skin
Purified water	up to 100	up to 100	up to 100
Sodium coco-sulfate, 100% powder	5.70	5.70	5.70
Decyl glucoside, 50% solution	5.00	5.00	5.00
Cocamidopropyl betaine, 40–45% solution	8.00	8.00	8.50
GLDA (glutamate diacetate tetrasodium salt), 47% solution	0.10	0.10	0.20
Sodium chloride	0.20	0.10–0.20	-
Glycerin	1.00	-	-
*Aloe barbadensis* leaf extract	0.10	0.10	0.10
Trimethylglycine	0.50	1.00	2.00
Glyceryl glucoside and glycerin	-	-	0.50
Polyquaternium-10 (Conditioning agent)	0.20	-	-
Citric acid monohydrate	0.50	0.4–0.6	0.4–0.6
Potassium sorbate, sodium benzoate	0.70	0.70	0.70
Fragrance and essential oils	0.55	0.55	-
Additional stabilizers of the formulations	0.001–0.1	0.001–0.1	0.001–0.1

## Data Availability

The data presented in this research are available on request from the corresponding author.
